# Medical Education, Pre- and Post-Pandemic Era: A Review Article

**DOI:** 10.7759/cureus.10775

**Published:** 2020-10-02

**Authors:** Aldanah Althwanay, Farah Ahsan, Federico Oliveri, Harshit K Goud, Zainab Mehkari, Lubna Mohammed, Moiz Javed, Ian H Rutkofsky

**Affiliations:** 1 Internal Medicine, California Institute of Behavioral Neurosciences & Psychology, Fairfield, USA; 2 Cardiology, California Institute of Behavioral Neurosciences & Psychology, Fairfield, USA; 3 Psychiatry/Neuroscience, California Institute of Behavioral Neurosciences & Psychology, Fairfield, USA

**Keywords:** medical education, virtual learning, traditional learning, pandemic era

## Abstract

A pandemic is the worldwide outbreak and spread of a disease. Although pandemics of influenza have occurred rarely, approximately once every few decades in more than three centuries, the outbreaks of H1N1 and H5N1 influenza, the severe acute respiratory syndrome (SARS), and most recently, the novel coronavirus disease (COVID-19) caused by severe acute respiratory syndrome coronavirus 2 (SARS-CoV-2), have necessitated the institution of protective and preventive measures such as school closure and mandatory quarantine of infected people, as social distancing is considered to be the most effective preventative strategy until the development of a vaccine, treatment, or both. The current pandemic has also resulted in a transformation in medical education for both undergraduate and postgraduate medical students. Clinical rotations for undergraduates have been suspended all over the world; inter-hospital residency rotations and combined teaching sessions have also been curtailed until further notice. During this most recent pandemic, a number of medical schools have immediately converted their whole clinical curriculum into online formats. Similarly, educational and clinical assessments have been converted into online assessments. However, as the pandemic eras tend to recur over time and epidemics will continue to break out, medical students and healthcare workers will remain susceptible to contagion. Hence, we need to adopt a new educational system that would be safe and sustainable in the long run.

## Introduction and background

Pandemic is defined as “an epidemic occurring worldwide, or over a very wide area, crossing international boundaries and usually affecting a large number of people” [1]. It affects every aspect of life, including education and the economy, as we know it. Pandemics of H1N1 and H5N1 influenza, the severe acute respiratory syndrome (SARS), and most recently, the novel coronavirus disease (COVID-19) caused by severe acute respiratory syndrome coronavirus 2 (SARS-CoV-2), have forced governments and authorities around the world to implement protective and preventive measures such as school closure and mandatory quarantine of cases as social distancing is considered to be the most effective preventative strategy until the development of a vaccine, treatment, or both [2-4].

While pandemics have historically created challenges, identifying these challenges is the first step in converting them into opportunities. Most medical schools were not prepared for such drastic changes, and some have struggled to come up with alternative methods to traditional learning, which comprises direct lecturing in classes, on-site clinical examinations with direct patient encounters, and clerking [5]. The majority of medical schools have learned from the SARS experience in 2003 and have developed pedagogical innovations that involve technology and simulation-based teaching, including online lectures, video clinical vignettes, virtual simulators, webcasting, and online chat-rooms to continue the educational process. On the other hand, the rest of the medical schools have closed their doors until further notice [6]. 

This virtual breakthrough has several benefits and drawbacks. Its success really depends on the level of advancement and the quality of the technological infrastructure of the facilities, which, unfortunately, are not standardized and are of poor quality in some institutions, especially in developing countries. Hence, we can clearly observe the inequity in the field of medical education. Other drawbacks include lack of real patient interactions, lack of direct interactions with peers, poor technical skills of educators, and most importantly, the psychological impact of quarantine and isolation. On the other hand, virtual learning has provided easy access to learning materials, the luxury of taking classes at home, safety, and decreased infectious hazards, in addition to the ease of communication means [7]. So, one might be tempted to think, can this virtual medical learning transform from a temporary emergent response method of learning into a permanent more sustainable method and replace the traditional in-person learning, especially since pandemics tend to recur over time and epidemics will continue to break out? [8]. Herein we review the most recent publications discussing the impact of the pandemic on the medical education among undergraduate and postgraduate medical students, virtual learning in comparison to the traditional learning, assessments and licensure during the pandemic, and the future of medical education in light of possible future outbreaks, in order to examine how to teach and assess medical students without the use of live patients, and to formulate contingency plans in the likely event that clinical teaching is again disrupted.

## Review

1. The shift in medical education during pandemics

The recent COVID-19 pandemic has resulted in a transformation in medical education for both undergraduate and postgraduate medical students, since social distancing is the most effective preventative strategy until the development of a vaccine, treatment, or both [4]. Hence, clinical rotations for undergraduates have been suspended; inter-hospital residency rotations and combined teaching sessions have also been curtailed until further notice. This has been especially detrimental for residents in surgical specialties as it has limited their hands-on surgical experience, case-log completions, and their clinical exposure, thereby disrupting their residency training [9]. However, such crises provide a great opportunity for medical educators to power the technology and to engage medical students and faculty in transforming the current pandemic-imposed remote medical education into an evidence-based paradigm [10]. Hence, most of the medical schools have quickly adapted to the online classes with the shifting of live clinical exposure to the virtual one using online lectures, video clinical vignettes, virtual simulators, webcasting, online chat-rooms, telehealth; even the research protocols have been modified and have become more flexible to adapt to the current conditions [5,9-11].

Although almost all medical schools have either shifted to virtual learning or suspended teaching until further notice, one might argue that epidemics will continue to break out and medical students will remain susceptible to contagion. Thus, it is inevitable for medical schools and hospitals to ensure that all medical students are well-trained in the use of personal protective equipment (PPE) and follow the infection control measures, rather than closing campuses and restricting their contact with real patients.

Similarly, from a pedagogical perspective of medical training, which states that medical students be treated as junior doctors and are an integral part of the healthcare team, students are subject to the same risks and duties. In fact, pandemic outbreaks will result in a generation of physicians and medical students that enter the profession with plenty of awareness and background about the occupational risks and possibility of mortality. However, we must remain mindful of the fact that medical students are not under the same contractual obligations as healthcare workers [12].

**Figure 1:**
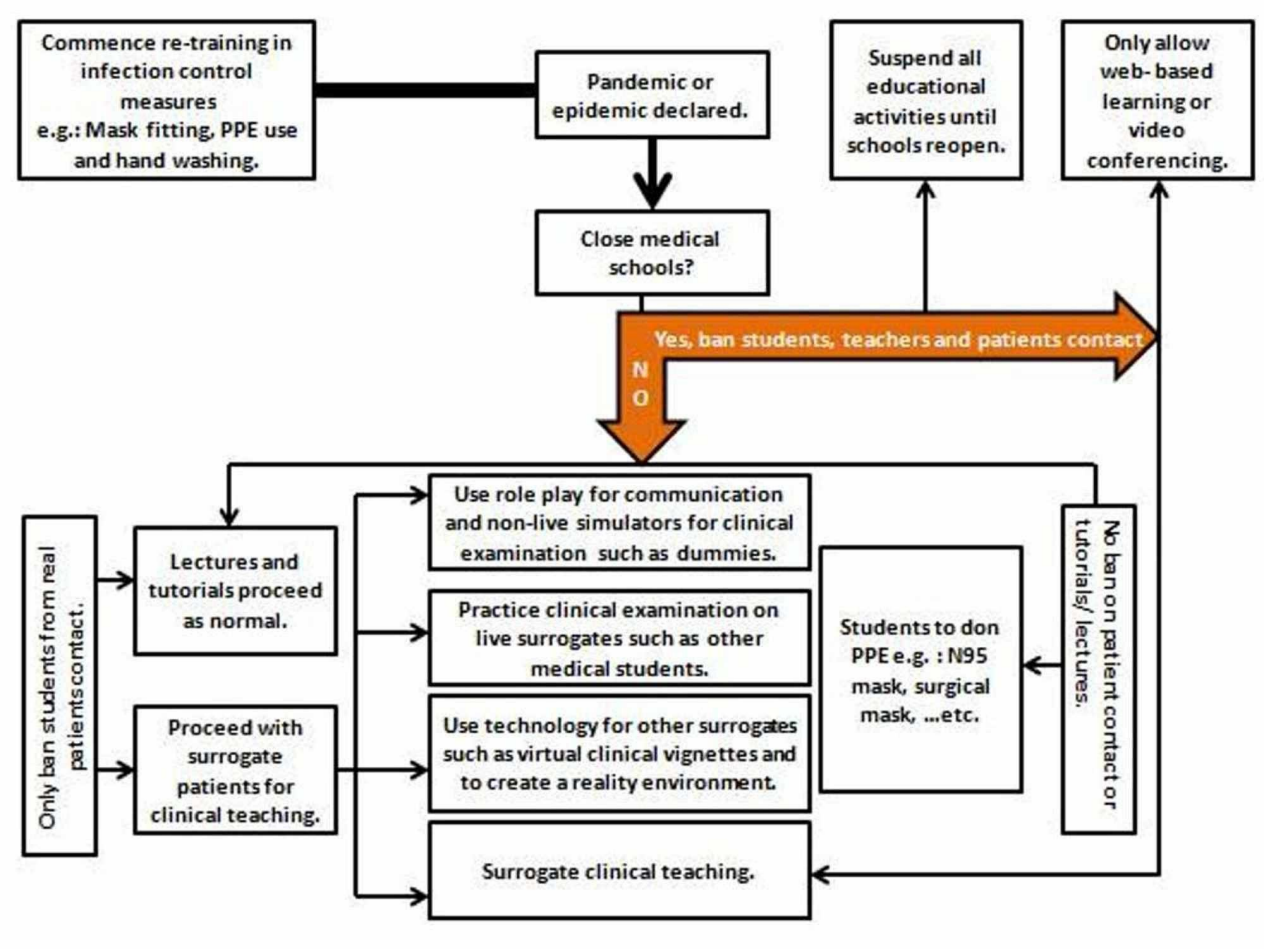
Decision tree for utilizing different teaching methods during a pandemic or epidemic Reproduced with permission from Lim E, Oh V, Koh D, Seet R: The Challenges of “Continuing Medical Education” in a Pandemic Era. Annals of the Academy of Medicine, Singapore. 2009; 38(8):724-726. Figure 1: Decision tree for utilizing different teaching methods during a pandemic or epidemic; p.725 PPE: personal protective equipment

Since medical students are not licensed or qualified to practice and employ their medical knowledge and clinical skills to treat patients yet, it might be argued that any participation in the care of a patient during a pandemic would primarily be for the students’ educational benefit rather than the provision of meaningful care for the patient. Therefore, the risks to the students' health might not outweigh the benefits. However, lots of medico-legal considerations such as malpractice insurance and health insurance issues might arise in such conditions even if they wanted to volunteer as skilled helpers during pandemics. Hence, medical education during pandemics remains controversial and more studies that measure the risks and benefits of continuing hospital teaching for students versus virtual simulated teaching are needed to reach a conclusive assessment. In addition, lots of aspects should be taken into account when making the decision as to which path should medical education pursue (Figure 1) [13-15]. However, despite students' safety being the priority, we truly believe that direct interaction with patients and bedside teachings are irreplaceable and integral components of medical education. Hence, a middle ground between at-home virtual learning and direct in-person teaching must be agreed upon.

2. Virtual learning versus traditional in-person learning

In traditional learning, pre-clinical education involves lectures, small group discussions, and laboratory sessions. Similarly, the traditional clinical phase of medical education consists of lectures and small group discussions in addition to in-hospital clinical rotations [16,17]. 

With the emergence of the current pandemic, medical schools have immediately converted their whole pre-clinical curriculum into online formats involving online lectures, webcasting, virtual group discussions, video-conferencing, and e-learning platforms that can be used to deliver lectures or tutorials via hand-held devices and laptops remotely [18-20]. However, such transition may be slower in areas where the technology infrastructure is still poor and underdeveloped, and online lectures still need to be prepared, especially when the majority of medical faculties have been redirected and forced to participate in fighting the pandemic. 

However, in order to evaluate this transition in an objective manner, we must take into account both the benefits and drawbacks of such a transition. Looking at the benefits of virtual learning, we have observed that online formats allow the students easy access to educational materials including the biggest international conferences as per their convenience, in their preferred environment in a consistent and seamless manner. In fact Chick et al. have reported that these innovative solutions utilizing technology may in fact help to bridge the educational gap for surgical residents during these unprecedented circumstances [20]. On the other hand, pitfalls of the new system include isolation due to shifting from the medical school setting to home setting, reduced discussions with peers, increased dependence on emails and issues with uninterrupted internet access, inability to define boundaries between work and home, and technophobia among the elder faculty [5,21]. Additionally, very few have incorporated the assessment component in these formats. Nevertheless, all these online formats require high-standard technology infrastructure, which costs an insane amount of money compared with regular traditional teaching [22,23].

Moreover, clinical experience is best acquired through real patient encounters at bedsides in hospitals. Not only does this experience provide medical students with first-hand experience of patients' clinical findings, but it also allows them to learn about the dynamics of patient interaction, psychology, and counseling. In addition, it provides specialty residents with an opportunity to revise their general medical and clinical skills, which can sometimes be forgotten after years of highly specialized training [20]. On the other hand, some studies have shown that simulated patients are as effective as real patients (if not more so) for teaching purposes [24,25].

**Figure 2:**
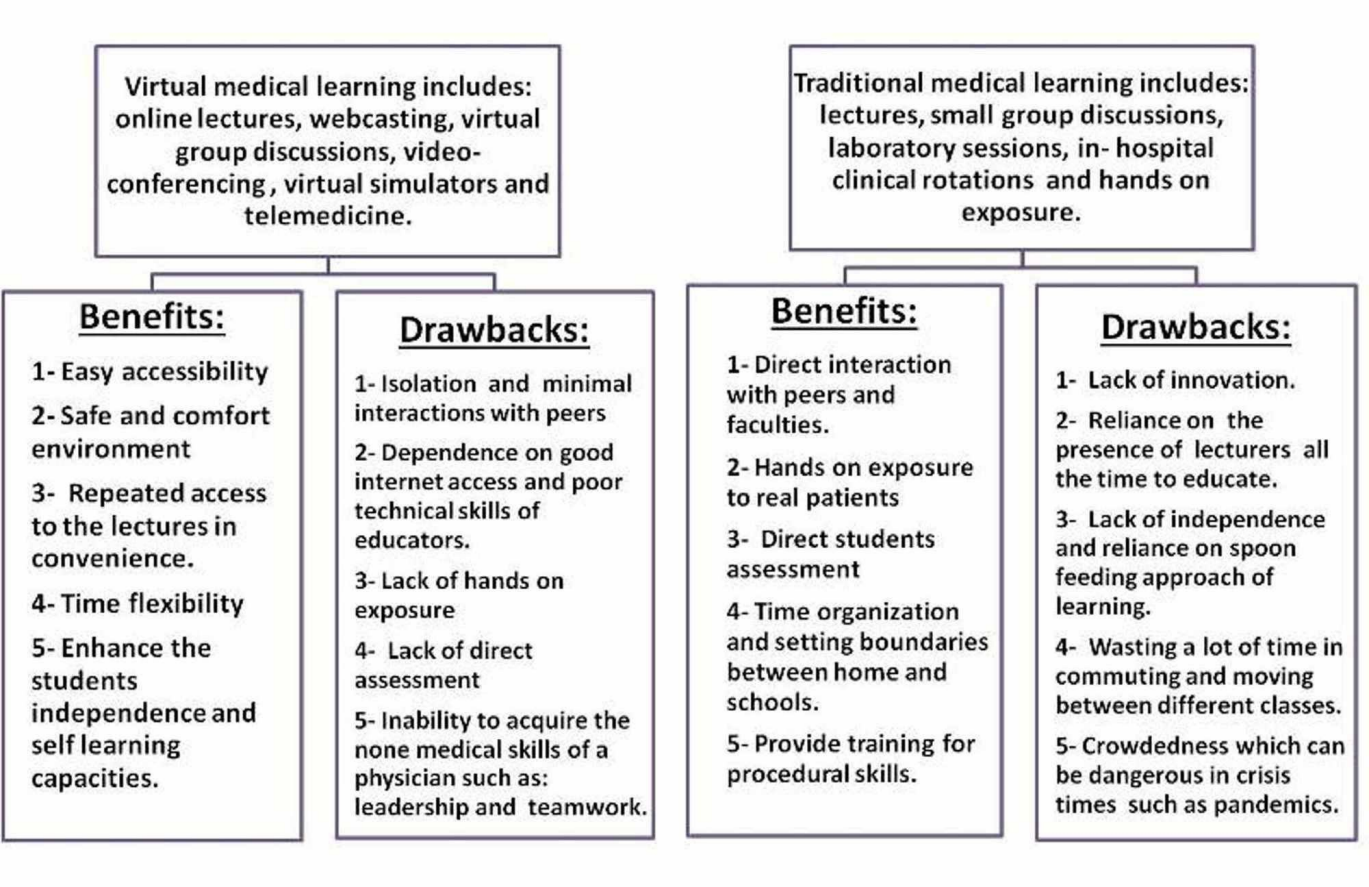
Comparison between traditional and virtual learning

Nevertheless, students' professional identity is developed through their medical teachers who are often perceived as role models. Additionally, those clinical teaching opportunities provide students with important lessons such as courage, empathy, leadership, and teamwork during their encounters (Figure 2) [8,26]. 

3. Assessments and licensure during the pandemic

In most medical schools, examinations have been delayed and postponed until further notice due to the pandemic. Such delays have affected the motivation of the students to study and have caused massive distress and uncertainty among others leading to serious psychological impacts such as depression and anxiety disorders [27]. The situation has been new and unprecedented for the majority of medical schools, and new methods of summative assessments have not been developed yet. Nevertheless, an important point to consider is the method of summative assessment to be employed while maintaining social distancing [8]. Back in 2003, during the SARS pandemic, the summative examinations at certain universities were conducted verbally via telephone-based viva voce and audio conferences [6]. However, during this most recent pandemic, the technology is much more advanced, and assessment methods have involved online web-based clinical case viva, demonstration of practical skills on virtual mannequins, and the use of digitized images for spotters as an online objective structured clinical examination (OSCE) [28]. However, an important concern to address would be whether the ongoing technology-based teaching/self-learning efforts are actually fulfilling the desired learning objectives or not [29]. Hence, formative assessments should be incorporated into such efforts. Moreover, self-assessment and peer assessment might be suitable methods in such situations and maybe encouraged while educators moderate the process [8].

In addition, rules should be set to ensure that the examination and assessment process is not violated or breached, despite being held at home; this can be done, for example, by using full-time proctors that moderate examinees while they take their exams [30,31]. 

Licensing examinations have also been affected by this pandemic. In the United States, for example, the clinical skills (CS) examination, which is an in-person interactive examination conducted in six centers across the states throughout the years since 2004, has been suspended since March 2020. Moreover, in May 2020, it was announced that it will be conducted as a telemedicine exam by August of the same year. However, it seems that this process will be lengthy, and it was suspended temporarily for 12-18 months and will resume afterward as a telemedicine exam for the first time ever [32,33]. Meanwhile, the Electronic Residency Application Service (ERAS®) portal submission has been postponed from September 15, 2020, to October 21, 2020, to allow candidates some extra time to compensate for the delays caused by the pandemic [34].

4. The future of medical education post-pandemic

When global pandemics emerge, they strike the whole world all of a sudden and affect every aspect of life with no one being prepared or ready for such a situation. The impact of the current pandemic on medical education has been unprecedented, far-reaching, and presents unique challenges to medical schools. It is frightening to consider that probably nothing will return to the way it was before even when the current pandemic eventually subsides. Therefore, we need to adopt a new educational system that would be safe, sustainable, and equipped for all kinds of unexpected scenarios in the future [35-37]. Hence, investing in virtual learning seems imperative right now as it seems destined to be the method of future medical education. Moreover, we need to create more online platforms that are easy to use for the students and faculty, as the ones used during crises so far have been good enough for emergency use only, and are not viable for long-term usage [38]. On the other hand, some medical educators have suggested combining the traditional face-to-face learning with online virtual learning as creating a combined method will offer the best of both methods individually and will be more durable and sustainable in the long run [10]. Interestingly, medical students have also been willing to participate in the decision-making process regarding the future of medical education, which should be encouraged in every educational institution since they are the ones who are predominantly impacted by the consequences of such decisions [39].

Moreover, since pandemic outbreaks are unusual circumstances, there are only a few publications in the literature that truly evaluate the impact of pandemics on the process of medical education, and the ones that are out there are based on limited institutional experiences, which is insufficient to generate appropriate generalizable recommendations on the future of medical education. However, in this review, we have collected the most recently published data about this topic, providing a summary of all the relevant work that is out there.

## Conclusions

In a nutshell, this review has summarized and presented the most recent findings and published evidence regarding medical education during pandemics and has concluded that the impact of a pandemic on medical education is controversial, unprecedented, challenging, and far-reaching. Therefore, the level of preparedness should be optimized and thought through in advance. Medical schools have immediately converted their clinical curriculum into online formats involving online lectures, webcasting, virtual group discussions, video-conferencing, and e-learning platforms that can be used to deliver lectures or tutorials remotely via hand-held devices and laptops as an emergency response for safety. However, since pandemic eras tend to recur over time and epidemics will continue to break out, putting students and patients at risk or proving deleterious in terms of patient care, investing in permanent mechanisms for virtual learning seems imperative to prevent the interruption of the educational process and since it seems destined to be the method of future medical education. However, we truly think that a middle ground should be reached between complete virtual learning at home and the direct in-person teaching in classrooms/hospitals rather than advocating for each option separately amid pandemics. Moreover, we endorse the view that medical students should be incorporated in the decision-making process regarding the future of medical education and should be having an active role as they are the ones who are predominantly impacted by the consequences of such decisions.
